# First person – Victoria Lockwood

**DOI:** 10.1242/bio.062859

**Published:** 2026-08-26

## Abstract

First Person is a series of interviews with the first authors of a selection of papers published in Biology Open, helping researchers promote themselves alongside their papers. Victoria Lockwood is first author on ‘Peak pressure experienced by the human hand during suspension and vertical climbing’, published in BiO. Victoria conducted the research described in this article while a PhD student in Professor Bernard Wood and Dr Carson Murray's lab at the Center for the Advanced Study of Human Paleobiology (CASHP) at George Washington University, Washington, USA. She is now a postdoctoral researcher in the lab of Dr Guillaume Daver at the Laboratory Paleontology Evolution Paleoecosystems Paleoprimatology, Université de Poitiers, France, investigating the level of behavioural resolution that it is possible to infer from the skeleton using a combined behavioural, biomechanical, and morphological research approach.

**Figure:**
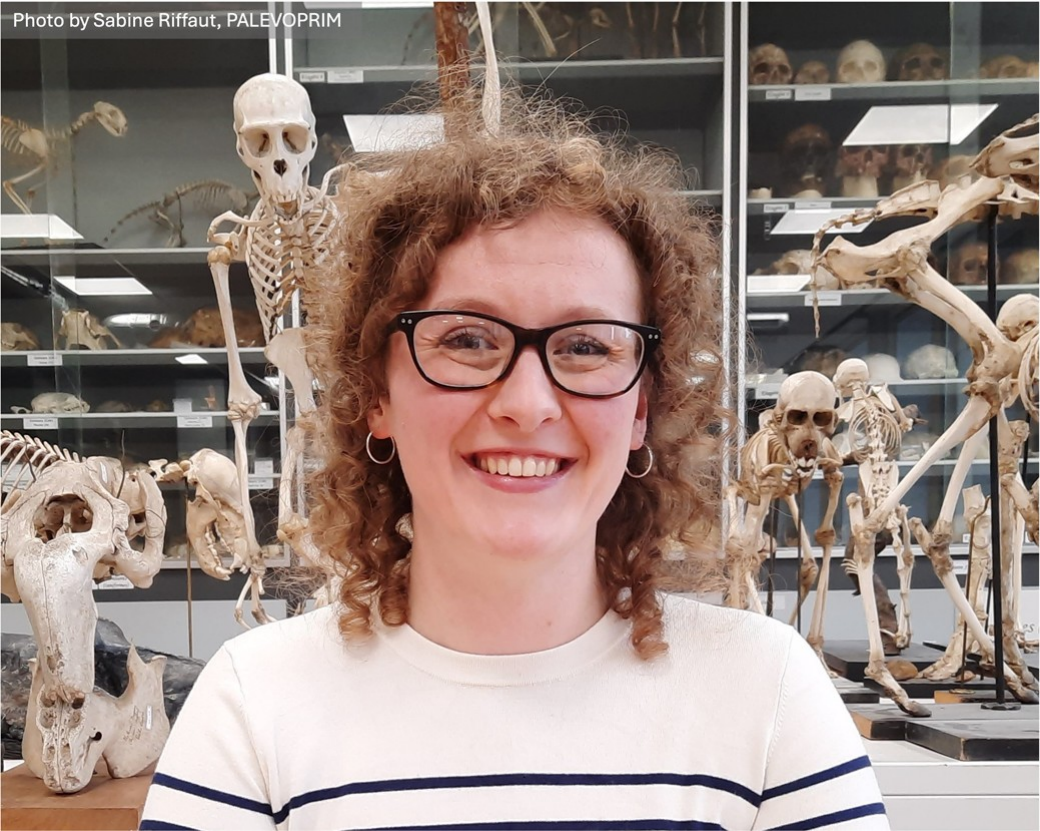
Victoria Lockwood

**Describe your scientific journey and your current research focus**

I am a palaeoanthropologist and primatologist who uses a combined behavioural, biomechanical, and morphological research approach to refine the level of behavioural resolution that can be obtained from fossil morphology. I particularly focus on the evolution of locomotion in addition to the manipulative abilities of the hand. Initially my research interests were with more modern human populations and forensic methodologies, I even explored primate brain evolution. However, when I was introduced to the hominin fossil record and the gaps in our knowledge when inferring how our ancestors and their relatives may have behaved, I was intrigued and wanted to find ways to improve our understanding and available methodologies. I started with the femur in relation to different types of primate locomotion and then I moved to the hand and the curious compromises its morphology presents between manipulative abilities and use in locomotion. Alongside this, and thanks to my mentor and co-supervisor Dr Carson Murray, I developed a collaboration with Gombe Stream Research Center to expand my understanding of extant primate behavioural variation and how it relates to the skeletal morphologies that I was observing in collections. Using perspectives from both palaeoanthropology and primatology has enabled me to develop research projects that start to untangle some of the behavioural uncertainties in hominins and I am excited to see where this takes me next.

**Who or what inspired you to become a scientist?**

My favourite word as a child was ‘why?’ and I was fortunate that my parents were willing to answer my constant questioning, particularly when observing animals or dinosaur museum exhibits. From those early questions I developed an interest in the past and how we could investigate what life was like across human history. I discovered ‘Time Team’, an archaeological TV programme, and through Dr (now Professor) Alice Roberts and her scientific curiosity I became fascinated by the amount of information that skeletons could provide about an individual's life. When the opportunity presented itself to keep asking questions and learn more about how we became human and what our evolutionary past was like, I was delighted to be able to follow that as a career path.

**How would you explain the main finding of your paper?**

Our study examined the peak pressure experienced by the hand and in what part of the hand the highest pressure was located during suspension and vertical climbing activities in humans. We found that for both suspension and vertical climbing the size of the support used impacted peak pressure values, with patterns aligning with the previous ergonomic literature for non-tree climbing activities (e.g. efficiently performing a task but with maximum comfort and safety). Whereas suspension and vertical climbing activities could be distinguished from each other via the location of peak pressure in the hand when using our experimental protocol.

**What are the potential implications of this finding for your field of research?**

The amount of pressure the hand experiences, amongst other things, can influence the shape of the skeleton in the hand, which is one of the key lines of evidence we use when interpreting how fossil hominins (human ancestors) might have moved in the trees. This is important as some of the hypotheses on the origins of bipedalism have arboreal components.

Knowing where in the hand the greatest pressure is experienced and how the amount of pressure varies with different sized supports – as trees have varying branch sizes – can aid the interpretation of hominin mosaic hand morphologies and the potential arboreal capabilities of hominins at a more detailed behavioural resolution than previously possible.

Our study is the first to present this data on the effects of support size and arboreal activity on how the human hand experiences peak pressure, so more investigation is needed especially in other hominids (great apes) to fully untangle the effects of different arboreal behaviours on hominin hand morphology.

**Figure:**
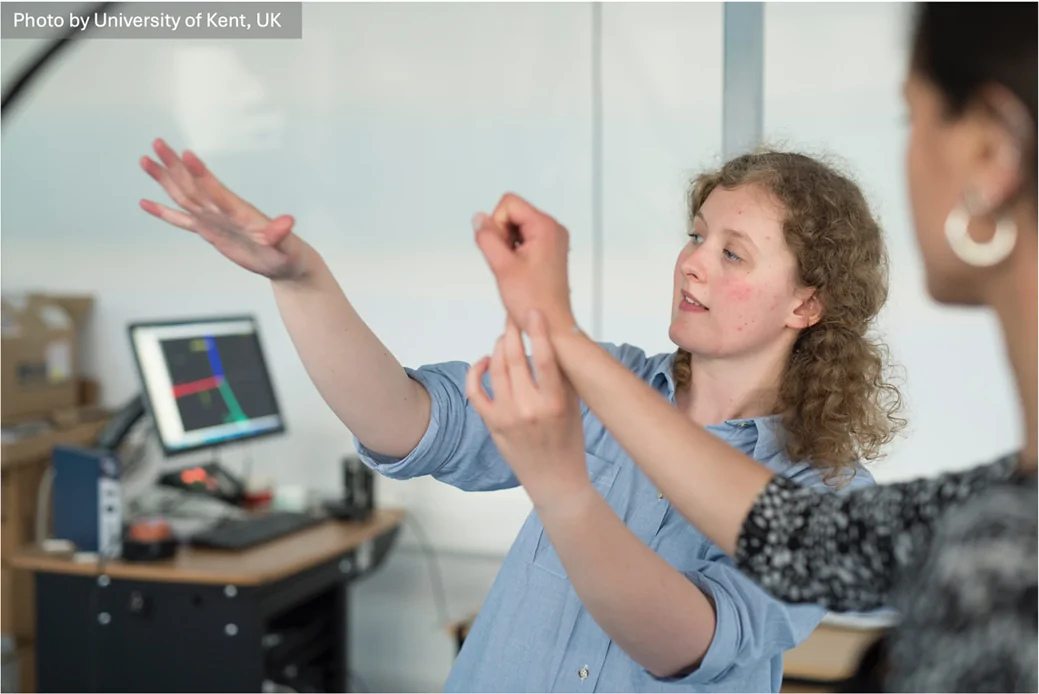
Victoria Lockwood demonstrates to an assistant how to place the hand during the arboreal locomotor activities.

**Which part of this research project was the most rewarding?**

The first time the synchronised pressure-kinematic protocol worked consistently. Generally, the pressure (Novel Pliance) and kinematic (Qualisys) technologies that we worked with are used separately within research, but to pinpoint the specific loading location in the hand at the moment of peak pressure required the systems to be synchronised. I spent months in the lab developing and refining the protocol and discussing with my collaborators until we had a version that worked consistently. Then it was on to data collection and the excitement of investigating our results as we mapped the 3D kinematic data on to the 2D pressure data and identified for the first time exactly where the human hand was experiencing peak pressure during arboreal suspension and vertical climbing.

**What do you enjoy most about being an early-career researcher?**

The enthusiasm of other early-career researchers for their specific research topics and the opportunities to discuss and learn new techniques and perspectives from them. I often find that talking to people from other research areas is a great way to ensure that you can clearly articulate your research approach and to see your research question with fresh eyes.

**What piece of advice would you give to the next generation of researchers?**

Don't be afraid to look at a research problem or question from a different angle, even if it seems a little unusual. Often there are other related disciplines that either have the information that you need to facilitate testing a hypothesis that currently seems a little out of reach or a research framework to help you untangle any knots you find as you develop your research questions. The phrase “I hadn't thought about it like that…” has the potential to lead to lots of intriguing possibilities.

**What's next for you?**

After finishing my PhD at CASHP, GWU, I completed a postdoctoral researcher position at the PALEVOPRIM lab at the University of Poitiers and in collaboration with La Vallée des Singes. During this postdoc I designed the Simulated Arboreal Bipedalism Research Equipment (SABRE) and collected the first gait parameter and 3D kinematic data for captive chimpanzee arboreal bipedalism. We recently published our first paper from this project (Lockwood et al., 2026), and there's more to follow! I am now looking for my next research opportunity, and I am always open to collaborations.
